# CircLDLR Promotes Papillary Thyroid Carcinoma Tumorigenicity by Regulating miR-637/LMO4 Axis

**DOI:** 10.1155/2021/3977189

**Published:** 2021-12-09

**Authors:** Yuan-ming Jiang, Wei Liu, Ling Jiang, Hongbin Chang

**Affiliations:** ^1^Department of Otolarynology, Wuhan No.1.Hospital, Wuhan 430030, China; ^2^General Surgery, Wuhan Hanyang Hospital, Wuhan 430000, China

## Abstract

**Background:**

Circular RNAs (circRNAs) have been reported to play important roles in the development and progression of papillary thyroid carcinoma (PTC). However, the function and molecular mechanism of circRNA low-density lipoprotein receptor (circLDLR) in the tumorigenesis of PTC remain unknown.

**Results:**

In this study, circLDLR was found to be markedly upregulated in PTC tissues and cell lines, and knockdown of circLDLR inhibited PTC cell proliferation, migration, and invasion but induced apoptosis *in vitro*. Moreover, circLDLR acted as a sponge for miR-637, and miR-637 interference reversed the anticancer effects of circLDLR knockdown on PTC cells. LMO4 was verified to be a target of miR-637; LMO4 upregulation abolished miR-637 mediated inhibition of cell growth and metastasis in PTC. Additionally, circLDLR could indirectly modulate LMO4 via acting as a sponge of miR-637 in PTC cells. Besides that, xenograft analysis showed that circLDLR knockdown suppressed tumor growth *in vivo* via regulating LMO4 and miR-637.

**Conclusion:**

Taken together, these results demonstrated that circLDLR promoted PTC tumorigenesis through miR-637/LMO4 axis, which may provide a novel insight into the understanding of PTC tumorigenesis and be useful in developing potential targets for PTC treatment.

## 1. Introduction

Thyroid cancer is the most common type of human malignancies in endocrine system and is histologically categorized into papillary, follicular, medullary, anaplastic, and poorly differentiated thyroid cancer [1, 2], among which, papillary thyroid carcinoma (PTC) is the most common type of thyroid carcinomas with a rising prevalence at an average annual rate of nearly 4% in recent years [1, 2]. Early PTC has relatively good survival, while the 5-year survival rate of PTC with advanced stage is only 59% [3]. Importantly, although great advance in the clinical treatment of PTC, including surgery and/or physiotherapy or chemotherapy, recurrence and metastasis still occurred with high rates [4]. Thus, it is of great significance to further identify the pathogenesis of PTC to develop novel effective treatments for PTC.

Circular RNAs (circRNAs) are one of stable and highly conservative noncoding RNA molecules with a covalently closed loop lacking the 5′-end cap and the 3′-end poly A tail [5, 6]. Increasing studies have shown the involvement of circRNAs in the tumorigenesis of many types of malignancy, such as gastric cancer [7], bladder cancer [8], hepatocellular carcinoma [9], and so on. Besides, previous studies also revealed that abnormal expression of circRNAs is drawn into the initiation and progression of PTC through by affecting the malignant behaviors of cancer cells [10, 11]. Circular RNA low-density lipoprotein receptor (circLDLR, hsa_circ_0003892) is a derived from LDLR gene with the length of 544 bp; it locates at chr19: 11230767-11238761. According to the analysis of GSE93522 dataset, the expression of circLDLR was found to be aberrantly upregulated in PTC. However, the biological functions of circLDLR in the progression of PTC remain unknown.

MicroRNAs (miRNAs) are one of small noncoding RNAs and can modulate specific gene expression programs by regulating posttranslational processes [9]. miRNAs have been documented to be involved in a variety of biological functions in cancers, thus affecting tumor progression [12]. Recently, Yuan et al. demonstrated that lncRNA HOTTIP promoted PTC progression by inducing cell malignant biological behavior via targeting miR-637 [13]. However, the action and molecular mechanism of miR-637 in PTC progression remain vague. Additionally, it is well known that miRNAs commonly exert their functions through targeting 3′-UTR of their target genes [14]. LIM-only protein 4 (LMO4) is a member of LIM domain only proteins (LMOs), which belongs to a subfamily of LIM-containing proteins [15]. Previous study showed LMO4 was increased in PTC and promoted cell growth, migration, and invasion via circBACH2/miR-139-5p/LMO4 axis [16]. Thus, it is not clear whether LMO4 is involved in PTC tumorigenesis as a target of miR-637.

In this study, we investigated the function of circLDLR in PTC carcinogenesis *in vitro* and *in vivo* and evaluated whether circLDLR exerted it biological functions via circLDLR/miR-637/LMO4 in regulating cell biological behaviors of PTC.

## 2. Materials and Methods

### 2.1. Clinical Specimens

Tumor tissues and para-tumor samples were collected from 45 PTC patients in Wuhan No.1.Hospital and then immediately stored at -80°C until RNA isolation. The screening of included PTC patients was shown in Fig. S1. Follow-up was conducted regularly every 3 months in the first 2 years after surgery and reduced to every 6 months from the third year. The last follow-up was performed in April 2019. We had obtained the agreement of the Ethics Committee of Wuhan No.1.Hospital, and written informed consent was collected form all patients before this study.

### 2.2. Cell Culture and Transfection

Human thyroid follicular epithelial cell line Nthy-ori 3-1 and human thyroid cancer cell lines (TPC-1, IHH-4) were purchased from Shanghai Academy of Life Science (Shanghai, China) and then were grown in RPMI-1640 medium (Gibco, Carlsbad, CA, USA) in accompany with 10% fetal bovine serum (FBS, Gibco) at 37°C with 5% CO_2_.

Small interfering RNA (siRNA) sequences targeting circLDLR (si-circLDLR, 5′-GTCCTCCCCATCGGACAAAGTdtdt-3′), pcDNA3.1-circLDLR overexpression vector (circLDLR), pcDNA3.1-LIM domain only 4 (LMO4) overexpression vector (LMO4), their negative control (si-NC: 5′-TTCTCCGAACGTGTCACGT-3′, circ-NC, vector), lentiviral particles stably expressing either short hairpin RNA- (shRNA-) targeting circLDLR (sh-circLDLR, 5′-CCGGGTCCTCCCCATCGGACAAAGTCTC GAGACTTTGTCCGATGGGGAGGACTTTTTG-3′) or a scrambled control sequence (sh-NC, 5′-TTCTCCGAACGTGTCACGTTCAAGAGACGTGACACGTTCGGA G AATTTTTT-3′) were designed and synthesized by Invitrogen (Carlsbad, CA, USA). The miR-637 mimic (sense, 5′-ACUGGGGGCUUUCGGGCUCUGCGU-3, antisense, 5′-GCAGAGCCC GAAAGCCCCCAGUUU-3′), miR-637 inhibitor (anti-miR-637) (5′-ACGCAGAGC CCGAAAGCCCCCAGU-3′), and their negative control (mimic control (NC): sense, 5′-UUCUCCGAACGUGUCACGUTT-3′, antisense, 5-ACGUGA CACGUUCGGAG AATT-3′; and inhibitor control (anti-NC): sense, 5′-CAGUACUU UUGUGUAGUCA A-3′) were achieved by RiboBio (Guangzhou, China). The transfection was conducted using Lipofectamine 3000 (Invitrogen) for 48 h.

### 2.3. Quantitative Real-Time Polymerase Chain Reaction (qRT-PCR)

The isolation of total RNA was performed with the help of Trizol reagent (Invitrogen). A PARIS Kit (Invitrogen) was used to define the subcellular localization of circLDLR according to the manufacturer's instructions. Complementary DNA (cDNA) generation was conducted using a High-Capacity cDNA Reverse Transcription Kit (Qiagen, Valencia, CA, USA), and then, qPCR was implemented by a miScript SYBR Green PCR Kit (Qiagen). The relative expression was detected using the 2^–*ΔΔ*Ct^ method with glyceraldehyde-3-phosphate dehydrogenase (GAPDH) or U6 small nuclear B noncoding RNA (U6) as an internal control. The PCR reaction program started at 95°C for 2 min, 40 cycles for 95°C for 10 s followed by 60°C for 30 s. The same experiment was repeated three times, and the average was taken. The primer sequences are as follows: circLDLR: F, 5′-CGTTGATGATATCTGTCCAAAATACTTTGTC-3′, R, 5′-CGATGGGGAGGACAATGGACAGAGCCCTC-3′; miR-637: F, 5′-ACUGGGGGCUUUCGGGCUCUGCGU-3′, R, 5′-ACGCAGAGCCCGAAAGCCCCCAGU-3′; LMO4: F, 5′-GGGATCGGTTCCACTACATCA-3′, R, 5′-GGTGACAATGAGGAAGGGCTA-3′; GADPH: F 5′-GAGAAACCTGCCAAGTATGATGAC-3′, R 5′-GGAGTTGCTGTTGAAGTCAC-3′, U6: F, 5′-CTCGCTTCGGCAGCACA-3′, R, 5′-AACGCTTCACGAATTTGCGT-3′.

### 2.4. RNase R Treatment

The RNA (2 *μ*g) was interacted with or without RNase R (3 U/mg, Qiagen) at 37°C for 20 min. Then, the resulting RNA was purified using an RNeasy MinElute Cleanup Kit (Qiagen) and subjected to qRT-PCR analysis. The results represent as the average of three independent replicates.

### 2.5. Cell Proliferation Analysis

For cell counting kit-8 (CCK-8) assay, TPC-1 and IHH-4 cells transfected with the assigned vector were placed in 96-well plates overnight (1 × 10^4^ cells/well) and then incubated with 10 *μ*L CCK-8 solution (Beyotime, Shanghai, China) for another 2 h at 24, 48, 72, or 96 h. Subsequently, the absorbance at 450 nm was measured. The experiment was repeated at least three times.

For colony formation assay, transfected cells (5000/well) with RPMI-1640 medium were seeded in 6-well plates, and the medium was replaced with new medium every 3 days. Finally, the typical images were photographed, and the number of visible colonies (≥50 cells) was counted after 14 days of incubation. The experiment was repeated three times.

### 2.6. Flow Cytometer

Transfected TPC-1 and IHH-4 cells (1 × 10^4^ cells/*μ*L) were collected and resuspended in binding buffer; then, 10 *μ*L Annexin V-FITC and propidium iodide (PI) were added into the cell suspension and interacted for 15 min under darkness. The apoptotic cells were evaluated by FACScan flow cytometer within 1 h. Three replicate wells were set in each group, and the experiment was repeated three times.

### 2.7. Transwell Assay

The invasion ability of cells was detected by a 24-well transwell chamber (8 *μ*m; Corning Costar, Cambridge, MA) precoated with Matrigel. Following transfection, cells suspended in serum-free medium (3 × 10^5^ cells/mL) were seeded in the upper chamber of transwell; then, 500 *μ*L complete medium mixed with 10% FBS was added into the lower chambers. 24 h later, invaded cells were counted by a microscope. The results represent as the average of three independent replicates.

### 2.8. Wound Healing Assays

Cell migration was analyzed by wound healing assays. Transfected cells were seeded into a 6-well plate (1 × 10^4^ cells/mL) overnight. Then, a sterile micropipette tip was utilized to create wounds in cell monolayers, followed by washing with PBS to remove free-floating cells and cell debris. After 24 h, representative images were captured, and the distance was measured to calculate cell migration. Experiments were performed three times, and the average was taken.

### 2.9. Western Blot

Proteins were extracted using RIPA lysis buffer (Beyotime, Beijing, China). Extractive proteins were separated by sodium dodecyl sulfate polyacrylamide gel electrophoresis and then shifted onto polyvinylidene fluoride membranes. The antibodies used in this study included cleaved-caspase3 (C-caspase3) (1 : 1000, ab2302, Abcam, Cambridge, MA, USA), matrix metallopeptidase 2 (MMP2) (1 : 5000; ab92536, Abcam), MMP9 (1 : 2000, ab38898, Abcam), LMO4 (1 : 5000, ab131030, Abcam), proliferating cell nuclear antigen (PCNA) (1 : 5000, ab29, Abcam), GAPDH (1 : 10000, ab181602, Abcam) primary antibodies (overnight, 4°C), and HRP-conjugated secondary antibody (1 : 3000, Sangon, Shanghai, China) (1 h, 37°C). The same experiment was repeated three times.

### 2.10. Dual-Luciferase Reporter Assay

The bioinformatics analysis was executed by the online database CircInteractome (https://circinteractome.nia.nih.gov/) or Targetscan (http://www.targetscan.org/vert_71/). Dual-luciferase reporter assay was used for validating the interaction between miR-637 and circLDLR or LMO4. Wild (wt) type and mutant (mut) circLDLR and LMO4 3′ UTR containing the predicted binding sites of miR-637 were cloned into the pmirGLO Vector (Promega, Shanghai, China) to generate pmirGLO-circLDLR-wt, pmirGLO-circLDLR-mut, pmirGLO-LMO4-wt, or pmirGLO-LMO4-mut. Then, these constructed vectors combined with miR-637 mimic or mimic control (miR-NC) were cotransfected into TPC-1 and IHH-4 cells using Lipofectamine 3000 (Invitrogen). 48 h later, the luciferase activity normalized to Renilla luciferase activity was examined by a dual luciferase assay kit (Promega). The same experiment was repeated three times.

### 2.11. RNA Immunoprecipitation (RIP) Assay

TPC-1 and IHH-4 cells were lysed RIP buffer (Millipore, Billerica, MA, USA), and cellular lysates were incubated with magnetic beads conjugated with human anti-Argonaute2 (Ago2) antibody (Millipore) or normal mouse IgG (Millipore) at 4°C for 4 h, followed by interaction with Proteinase K to digest the protein. Finally, immunoprecipitated RNA was extracted, and purified RNA was subjected to qRT-PCR analysis. Analyses were performed in triplicates.

### 2.12. Xenograft Experiments *In Vivo*

BALB/c nude mice (*N* = 6, 4-6 weeks old) were obtained from National Laboratory Animal Center (Beijing, China). 1 × 10^6^ TPC-1 cells stably transfected with sh-circLDLR or sh-NC were subcutaneously injected into the flanks of the nude mice. Tumor size was determined each week to calculate tumor volume. At day 35, mice were euthanized, and tumors of each group were weighed and harvested for molecular analysis. Mice were killed by cervical dislocation after deep anesthesia with 2% isoflurane. All experiments were done in Wuhan No.1.Hospital and approved by the Animal Research Committee of Wuhan No.1.Hospital.

### 2.13. Statistical Analysis

Data of at least three experiments were shown as mean values with standard deviation (SD). All statistical analyses were performed using the GraphPad Prism 7 software. Statistical differences between groups were analyzed using Student's *t*-test, one-way or two-way analysis of variance (ANOVA) followed by Tukey's test as appropriate. The correlation analysis was carried out with Pearson correlation analysis. *P* value < 0.05 (∗), *P* value < 0.01 (∗∗), or *P* value < 0.001 (∗∗∗) was considered as statistically significant.

## 3. Results

### 3.1. CircLDLR Expression in PTC and Its Correlation with Overall Survival

**Figure 1:**
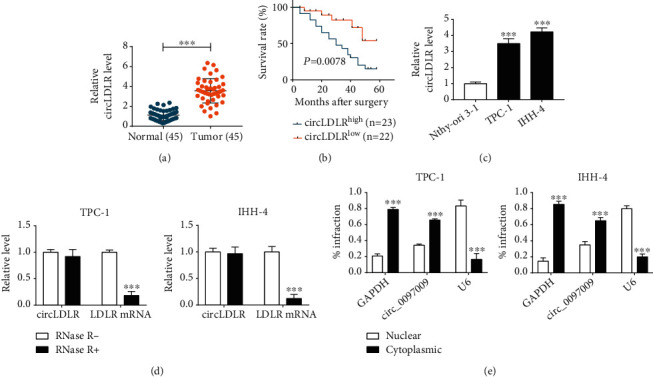
CircLDLR expression in PTC and its correlation with overall survival. (a) qRT-PCR analysis of circLDLR expression in 45 PTC tissues and matched normal tissues. (b) Kaplan-Meier survival curve analysis for the correlation between circLDLR expression and overall survival rate in PTC. (c) qRT-PCR analysis of circLDLR expression in PTC cell lines (TPC-1 and IHH-4) and normal Nthy-ori 3-1 cell lines. (d) qRT-PCR analysis of circLDLR and LDLR mRNA expression in TPC-1 and IHH-4 cells in the presence or absence of RNase R (e) Nuclear and cytoplasmic fraction experiment displaying the location of circLDLR in TPC-1 and IHH-4 cells. ^∗∗∗^*P* < 0.001.

The expression of circLDLR in 45 PTC tissues and matched normal tissues was detected, and qRT-PCR analysis displayed that circLDLR was remarkably elevated in tumor tissues relative to those in normal tissues (Figure 1(a)). Then, the correlations of circLDLR expression and special clinicopathological parameters of PTC were analyzed; it was proved that higher circLDLR expression was correlated with advanced TNM stages, tumor size, and lymph node metastasis (Table 1, *P* < 0.05). Besides that, patients with PTC were divided into two groups depending on the median level of circLDLR expression, and we found that patients in the high circLDLR group had a significantly shorter overall survival than those in the low circLDLR group (Figure 1(b)). Then, the expression of circLDLR in cells was measured; as expected, circLDLR also was higher in PTC cell lines (TPC-1 and IHH-4) than those in normal Nthy-ori 3-1 cell lines (Figure 1(c)). Afterwards, the stability of circLDLR was investigated. Total RNA from proliferating TPC-1 and IHH-4 cells was treated with RNase R; by contrast with linear LDLR mRNA, circLDLR could resistant to the degradation by RNase R, indicating circLDLR stably functioned as a typical circRNA (Figure 1(d)). Besides that, qRT-PCR indicated that circLDLR was distributed mainly in the cytoplasm (Figure 1(e)). These data confirmed that circLDLR expression was elevated in PTC, and high circLDLR predicted poor overall survival in patients with PTC.

### 3.2. CircLDLR Knockdown Suppresses Cell Malignant Phenotypes in PTC

**Figure 2:**
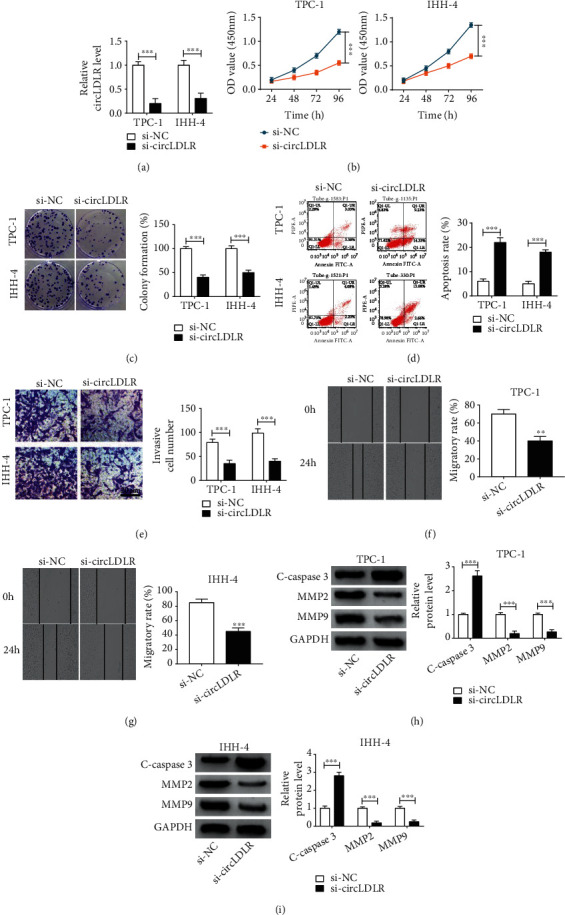
CircLDLR knockdown suppresses cell malignant phenotypes in PTC. TPC-1 and IHH-4 cells were transfected with si-circLDLR or si-NC. (a) qRT-PCR analysis of circLDLR expression in TPC-1 and IHH-4 cells after transfection. (b, c) The proliferation analysis of TPC-1 and IHH-4 cells using CCK-8 assay and colony formation assay. (d) Flow cytometry analysis of apoptosis of TPC-1 and IHH-4 cells. (e–g) The analysis of invasion and migration abilities of TPC-1 and IHH-4 cells using transwell assay and wound healing assay. (h, i) Western blot analysis of C-caspase3, MMP2, and MMP9 protein levels. ^∗∗^*P* < 0.01, ^∗∗∗^*P* < 0.001.

To explore the function of circLDLR in PTC, TPC-1 and IHH-4 cells were transfected with si-circLDLR or si-NC; then, qRT-PCR analysis showed that circLDLR expression was significantly reduced by si-circLDLR as expected (Figure 2(a)). Subsequently, CCK-8 assay indicated that circLDLR knockdown inhibited the proliferation of TPC-1 and IHH-4 cells (Figure 2(b)); similarly, colony formation analysis also revealed that circLDLR silencing decreased the number of colonies formed in TPC-1 and IHH-4 cells (Figure 2(c)). Conversely, the apoptosis of TPC-1 and IHH-4 cells was induced by circLDLR knockdown (Figure 2(d)). Meanwhile, transwell assay suggested that the invasion ability of TPC-1 and IHH-4 cells was suppressed by circLDLR downregulation (Figure 2(e)); besides, wound-healing assay showed that circLDLR knockdown also inhibited the migration of TPC-1 and IHH-4 cells (Figures 2(f) and 2(g)). Moreover, the expression of C-caspase3 was upregulated, while MMP2 and MMP9 expression was downregulated by circLDLR knockdown in TPC-1 and IHH-4 cells (Figures 2(h) and 2(i)). Taken together, circLDLR knockdown suppressed PTC progression.

### 3.3. CircLDLR Functions as an Efficient miR-637 Sponge in PTC Cells

**Figure 3:**
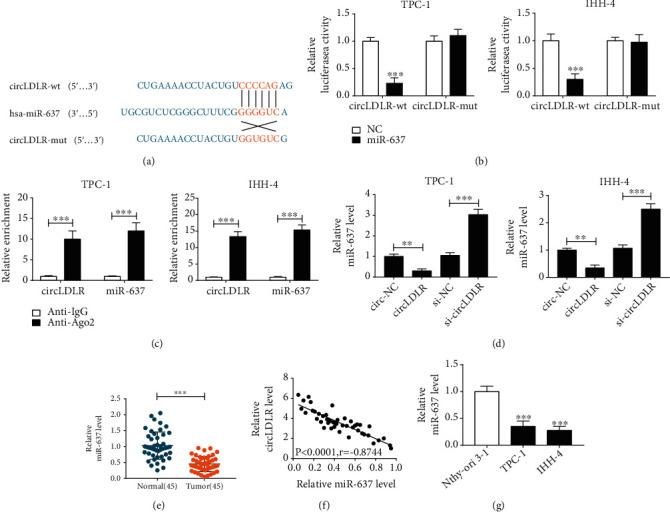
CircLDLR functions as an efficient miR-637 sponge in PTC cells. (a) Potential binding sites of circLDLR in miR-637 sequences. (b, c) Interaction analysis between circLDLR in miR-637 in TPC-1 and IHH-4 cells using the dual-luciferase reporter assay and RIP assay. (d) qRT-PCR analysis of miR-637 expression in TPC-1 and IHH-4 cells transfected with circ-NC, circLDLR, si-circLDLR, or si-NC. (e) Levels detection of miR-637 in 45 PTC tissues and matched normal tissues with qRT-PCR. (f) The correlation analysis between circLDLR and miR-637 using Pearson correlation analysis. (g) qRT-PCR analysis of miR-637 expression in PTC cell lines (TPC-1 and IHH-4) and normal Nthy-ori 3-1 cell lines. ^∗∗^*P* < 0.01, ^∗∗∗^*P* < 0.001.

It has been reported that circRNAs can act as miRNA sponges to modulate the expression of downstream mRNAs [17]. Thus, whether circLDLR enhanced the malignant biological behaviors of PTC cell by sponging miRNAs was explored. According to prediction of CircInteractome, seven miRNAs with high score were selected, and we found that circLDLR knockdown significantly led to an increase of miR-637 expression in TPC-1 cells (Fig. S2). Thus, we hypothesized that miR-637 might be a target of circLDLR. The binding sites of miR-637 on circLDLR are shown in Figure 3(a). Then, the dual luciferase reporter assay exhibited that miR-637 overexpression dramatically reduced the luciferase activity of the circLDLR-wt group in TPC-1 and IHH-4 cells but not the circLDLR-mut group (Figure 3(b)). In the meanwhile, we discovered that circLDLR and miR-637 pulled down by Anti-Ago2 was predominantly enriched in the Ago2 overexpression group compared with the control Anti-IgG group in TPC-1 and IHH-4 cells (Figure 3(c)). These results confirmed that circLDLR targeted miR-637 in PTC. Additionally, qRT-PCR analysis indicated that miR-637 expression was inhibited by circLDLR overexpression but was elevated by circLDLR downregulation in TPC-1 and IHH-4 cells (Figure 3(d)). Subsequently, the expression of miR-637 in PTC was detected, and miR-637 was decreased in PTC tissues (Figure 3(e)) and was negatively correlated with circLDLR (Figure 3(f)). Also, miR-637 had a low expression in TPC-1 and IHH-4 cells compared with the normal Nthy-ori 3-1 cell lines (Figure 3(g)). Therefore, these results indicated that miR-637 was a target of circLDLR and might be associated with the development of PTC.

### 3.4. MiR-637 Interference Reverses the Antitumor Effects Induced by si-circLDLR in PTC Cells

**Figure 4:**
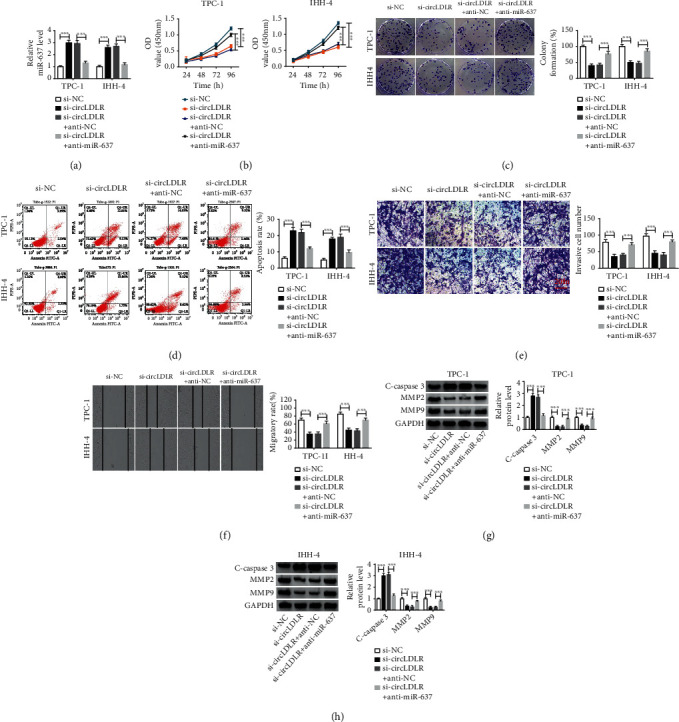
miR-637 interference reverses the antitumor effects induced by si-circLDLR in PTC cells. TPC-1 and IHH-4 cells were transfected with si-NC, si-circLDLR, si-circLDLR + anti-NC, or si-circLDLR + anti-miR-637. (a) qRT-PCR analysis of miR-637 expression after transfection. (b, c) The proliferation analysis of TPC-1 and IHH-4 cells using CCK-8 assay and colony formation assay. (d) Apoptosis analysis of TPC-1 and IHH-4 cells using flow cytometry. (e, f) Analysis of TPC-1 and IHH-4 cell migration and invasion with transwell assay and wound healing assay. (g, h) Levels detection of C-caspase3, MMP2, and MMP9 protein levels using western blot. ^∗∗∗^*P* < 0.001.

We then investigated whether circLDLR promoted the progression of PTC by interacting with miR-637. TPC-1 and IHH-4 cells were transfected with si-NC, si-circLDLR, si-circLDLR + anti-NC, or si-circLDLR + anti-miR-637, and qRT-PCR analysis showed that miR-637 inhibition attenuated si-circLDLR-induced elevation of miR-637 in TPC-1 and IHH-4 cells (Figure 4(a)), suggesting the success of transfection. After that, rescue assay was conducted, and results showed miR-637 interference reversed circLDLR knockdown-mediated impairments of the proliferation (Figures 4(b) and 4(c)), invasion (Figure 4(e)) and migration (Figure 4(f)), and promotion of apoptosis (Figure 4(d)) in TPC-1 and IHH-4 cells. Accordingly, western blot analysis also confirmed miR-637 inhibition could partly abate si-circLDLR-mediated migration and invasion inhibition and apoptosis enhancement, evidenced by the change in C-caspase3, MMP2, and MMP9 protein levels in TPC-1 and IHH-4 cells (Figures 4(g) and 4(h)). In all, we verified that circLDLR promoted PTC progression partly by downregulating miR-637.

### 3.5. LMO4 Is a Target of miR-637 and circLDLR Regulates LMO4 via miR-637 in PTC Cells

**Figure 5:**
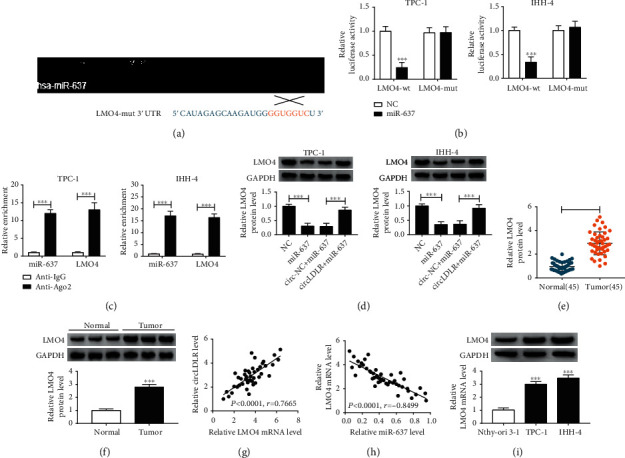
LMO4 is a target of miR-637, and circLDLR regulates LMO4 via miR-637 in PTC cells. (a) The predicted binding sites of miR-637 on LMO4 sequences. (b) Luciferase activity detection in TPC-1 and IHH-4 cells cotransfected with the reporter plasmid and the indicated miRNAs using the dual-luciferase reporter assay. (c) qRT-PCR analysis of miR-637 and LMO4 expression in TPC-1 and IHH-4 cells pulled down by Anti-Ago2 or Anti-IgG. (d) Western blot analysis of LMO4 level in TPC-1 and IHH-4 cells transfected with NC, miR-637, circ-NC + miR-637, and circLDLR + miR-637. (e, f) Level detection of LMO4 in 45 PTC tissues and matched normal tissues with qRT-PCR and western blot. (g, h) Correlation analysis between LMO4 and circLDLR or miR-637 in PTC tissues with Pearson correlation analysis. (i) Level detection of LMO4 in PTC cell lines (TPC-1 and IHH-4) and normal Nthy-ori 3-1 cell lines with western blot. ^∗∗∗^*P* < 0.001.

The related target genes of miR-637 were further predicted. According to the prediction of Targetscan program, LMO4 was found that might be a target of miR-760 with putative binding sites (Figure 5(a)). Then, a reduction of luciferase activity in TPC-1 and IHH-4 cells cotransfected with LMO4-wt and miR-637 mimic was detected (Figure 5(b)). Moreover, RIP assay displayed that LMO4 and miR-637 expression was notably enriched in Ago2 immunoprecipitates relative to the control IgG immunoprecipitates (Figure 5(c)). These data revealed that LMO4 was a target of miR-637. Afterwards, we discovered that LMO4 expression was decreased by miR-637 overexpression, and miR-637 overexpression reversed circLDLR-induced increase of LMO4 level in TPC-1 and IHH-4 cells (Figure 5(d)). Thus, a circLDLR/miR-637/LMO4 axis in PTC was identified. After that, the expression of LMO4 was analyzed; we found that LMO4 expression was upregulated in PTC tumor tissues at mRNA and protein levels (Figures 5(e) and 5(f)). Besides that, LMO4 was positively correlated with circLDLR (Figure 5(g)), while negatively correlated with miR-637 (Figure 5(h)) in PTC tissues, further suggesting the relationship among circLDLR, miR-637, and LMO4 in PTC. Similarly, LMO4 was also elevated in PTC cell lines (Figure 5(i)). These results suggested that miR-637 targeted LMO4 and circLDLR regulated LMO4 via miR-637 in PTC cells.

### 3.6. MiR-637 Suppresses Cell Malignant Phenotypes in PTC Cells by Targeting LMO4

**Figure 6:**
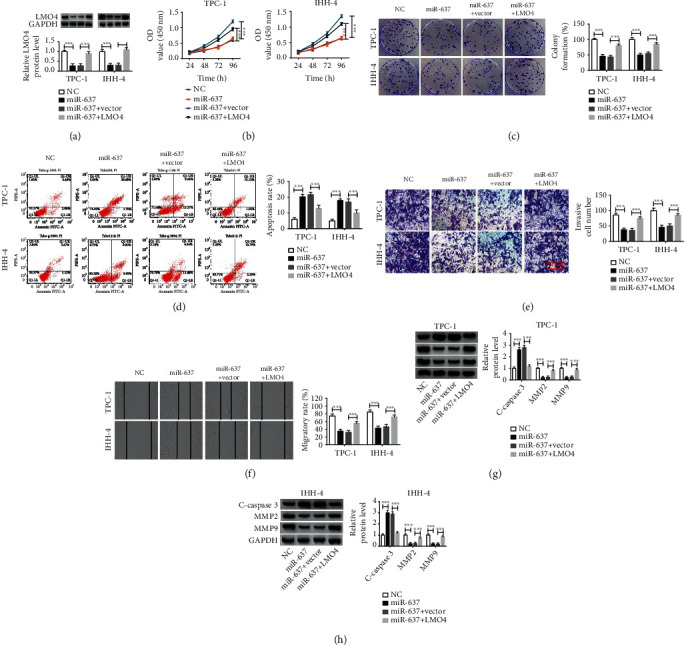
MiR-637 suppresses cell malignant phenotypes in PTC cells by targeting LMO4. TPC-1 and IHH-4 cells were transfected with NC, miR-637, miR-637 + vector, or miR-637 + LMO4. (a) Western blot analysis of LMO4 expression in TPC-1 and IHH-4 cells after transfection. (b, c) The proliferation analysis of TPC-1 and IHH-4 cells using CCK-8 assay and colony formation assay. (d) Flow cytometry analysis of apoptosis of TPC-1 and IHH-4 cells. (e, f) The analysis of invasion and migration abilities of TPC-1 and IHH-4 cells using transwell assay and wound healing assay. (g, h) Western blot analysis of C-caspase3, MMP2, and MMP9 protein levels. ^∗∗∗^*P* < 0.001.

Given the axis of circLDLR/miR-637/LMO4 in PTC cells, we further studied whether LMO4 was involved in the action of circLDLR/miR-637 axis in PTC progression. TPC-1 and IHH-4 cells were transfected with NC, miR-637, miR-637 + vector, or miR-637 + LMO4, and then qRT-PCR analysis showed that LMO4 overexpression rescued miR-637 mimic-induced decrease of LMO4 in TPC-1 and IHH-4 cells (Figure 6(a)). Then, functional experiments were conducted. Results exhibited that miR-637 re-expression impaired the proliferation (Figures 6(b) and 6(c)), invasion (Figure 6(e)), and migration (Figure 6(f)) but induced apoptosis (Figure 6(d)) in TPC-1 and IHH-4 cells. Besides that, miR-637 overexpression led to the increase of C-caspase3 and decrease of MMP2 and MMP9 in TPC-1 and IHH-4 cells (Figures 6(g) and 6(h)). Thus, miR-637 inhibited PTC progression. However, rescue assay showed that the antitumor functions of miR-637 were significantly reversed by LMO4 overexpression in TPC-1 and IHH-4 cells (Figures 6(b) and 6(h)). Altogether, miR-637 suppressed PTC progression partly by targeting LMO4.

### 3.7. CircLDLR Knockdown Impedes PTC Growth *In Vivo*

**Figure 7:**
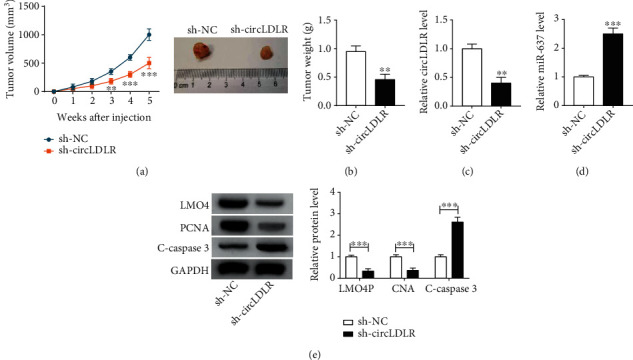
CircLDLR knockdown impedes the growth of xenograft tumors *in vivo*. TPC-1 cells stably transfected with sh-circLDLR or sh-NC were subcutaneously injected into the flanks of the nude mice to establish xenograft models. (a, b) The detection of the size and weight of xenograft tumors. (c, d) qRT-PCR analysis of circLDLR and miR-637 levels in tumor masses. (e) Western blot analysis of LMO4, PCNA, and C-caspase3 levels in in tumor masses. ^∗∗^*P* < 0.01, ^∗∗∗^*P* < 0.001.

To investigate the functions of circLDLR *in vivo*, a xenograft tumor model was established. As shown in Figures 7(a) and 7(b), circLDLR knockdown suppressed the growth of xenograft tumors, evidenced by the smaller size and lighter weight of tumors in the sh-circLDLR group. In addition, knockdown of circLDLR decreased the levels of circLDLR and LMO4 (Figures 7(c) and 7(e)) but increased the level of miR-637 (Figure 7(d)) in xenograft tumors. Furthermore, western bolt analysis showed that PCNA was decreased, but C-caspase3 was increased in the xenograft tumors of the sh-circLDLR group compared with the sh-NC group, furthering revealing circLDLR silencing suppressed tumor growth *in vivo*. Collectively, circLDLR silencing hindered tumor growth *in vivo* via regulating miR-637 and LMO4.

## 4. Discussion

PTC is the most frequent endocrine malignancy and generally has a good prognosis. Nevertheless, accumulating evidence has exhibited that early stage extradural invasion, lymph node metastasis, distant metastases, and recurrence occasionally occur in some cases, which lead to a poor prognosis, even death in PTC patients [14, 18]. Up to date, increasing researches have reported the implication of circRNAs in the development and progression of cancers through modulating diverse cellular processes [16, 19, 20]. In PTC, some circRNAs have also been indicated to be implicated in cancer progression. For example, Wei et al. found that circZFR promoted cell proliferation and invasion in PTC via upregulating C8orf4 expression through miR-1261 [21]. Bi et al. revealed that circRNA_102171 contributed to the progression PTC by activating CTNNBIP1/Wnt/*β*-catenin [22]. Thus, circRNAs may be promising candidates for controlling the progression of PTC.

According to the analysis of GSE93522 dataset, the expression of circLDLR was found to be aberrantly upregulated in PTC. Importantly, we also observed that circLDLR was elevated in clinical PTC tissues and cell lines, and high circLDLR expression predicted poor prognosis. However, the biological function of circLDLR in PTC tumorigenesis was not reported yet. Sustained proliferation and metastatic progression are the major drivers of lethality in cancers, and metastasis accounts for the vast majority of patient mortality [28-30]. Thus, the role of circLDLR in PTC cell growth and metastasis was investigated. We found that knockdown of circLDLR inhibited PTC cell proliferation, invasion, and migration but facilitated apoptosis *in vitro*. Moreover, clinical relevance of circLDLR was validated by the observed suppression in ccRCC tumorigenic in nude mice after circLDLR silencing. Altogether, we demonstrated that circLDLR siRNA might be a promising molecule for the treatment of PTC.

It has been identified that circRNAs can function as miRNA sponges to modulate gene expression through abolishing the activity of miRNAs [17]. Subsequently, whether circLDLR served it biological function by acting as miRNA sponge was investigated. Through bioinformatics analysis and interaction analysis, we confirmed that miR-637 was a target of circLDLR in PTC cells. MiR-637 is a well-recognized tumor suppressor. For instance, miR-637 suppressed hepatoma cell viability and invasion via targetedly degrading the expression of AKT1 [23]. MiR-637 decrease was related to poor overall survival and accelerated malignant properties of glioma cells by targeting AKT1 [24]. However, the function of miR-637 in PTC remains unclear. In our work, miR-637 was downregulated in PTC tissues and cell lines; then, we discovered that restoration of miR-637 suppressed cell malignant phenotypes in PTC, thereby hindering cancer progression. After that, rescue assay suggested miR-637 inhibition reversed the inhibitory effects of circLDLR knockdown on PTC tumorigenesis. Therefore, the role of circLDLR/miR-637 axis in the development of PTC was firstly identified.

Recently, growing evidence has identified the involvement of LMO4 in the tumorigenesis. LMO4 has been identified to mediate interactions between multiprotein complexes and DNA, which modulates the expression of genes participated in modulating various biological processes, like cell survival and mammalian development [25]. Additionally, Wang et al. revealed that LMO4 facilitated tumor cell malignant phenotypes in gastric cancer via activating PI3K-Akt-mTOR signaling [26]. Zhou et al. demonstrated that LMO4 suppressed p53-mediated repression of cell proliferation in breast cancer by blocking p53 [27]. Thus, dysregulation of LMO4 plays an important role in the carcinogenesis of cancer. In this study, the expression of LMO4 was found to be elevated in PTC; besides, LMO4 was confirmed to be a target of miR-637, and LMO4 upregulation attenuated the antitumor effects of miR-637 in PTC cells. Thus, the anticancer function of the miR-637/LMO4 pathway in PTC was demonstrated. More importantly, this study also detected that circLDLR could indirectly regulate LMO4 expression via miR-637 in PTC.

**Figure 8:**
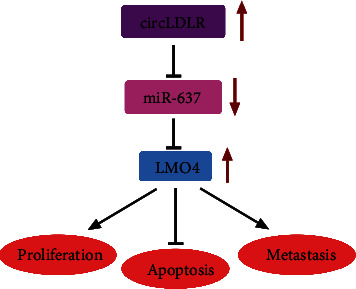
The schematic diagram illustrates circLDLR/miR-637/LMO4 axis in PTC tumorigenesis. CircLDLR promotes proliferation and metastasis and suppresses apoptosis of PTC through miR-637/LMO4 axis.

In summary, our findings firstly proved that circLDLR acted as an oncogene to promote PTC progression by promoting cancer cell malignant biological behaviors via miR-637/LMO4 pathway (Figure 8). This study offers an improved understanding of the pathogenesis of PTC and reveals a novel regulatory pathway, which may be targeted for therapeutic benefits. Nevertheless, further investigations are needed to probe whether circLDLR/miR-637/LMO4 can affect the phenotypes of normal cells, thus uncovering the clinical applicability of these molecules as the anticancer drug.

## Figures and Tables

**Table 1:** Correlation between circLDLR expression and clinicopathological parameters of papillary thyroid carcinoma patients (*n* = 45).

Clinical feature		circLDLR		
	n	High	Low	*P* value
Age				0.449
≥45	24	11	13	
<45	21	12	9	
Gender				0.884
Man	23	12	11	
Woman	22	11	11	
TNM stage				0.011
I/II	18	5	13	
III/IV	27	18	9	
Tumor size (cm^3^)				<0.001
≥3	24	18	6	
<3	21	5	16	
Lymph node metastasis				0.005
N0	23	7	16	
N1	22	16	6	

## Data Availability

No data were used to support this study.

## Supplementary Materials

Supplementary Materials Figure S1: the diagram of the screening of included PTC patients. Figure S2: the selection of the potential targeted miRNAs of circLDLR.
