# Supplementary figures and images for: CircLDLR Promotes Papillary Thyroid Carcinoma Tumorigenicity by Regulating miR-637/LMO4 Axis
Source: Dis Markers. 2021 Dec 9;2021:3977189. doi: 10.1155/2021/3977189 (PMC8677406; doi:10.1155/2021/3977189)

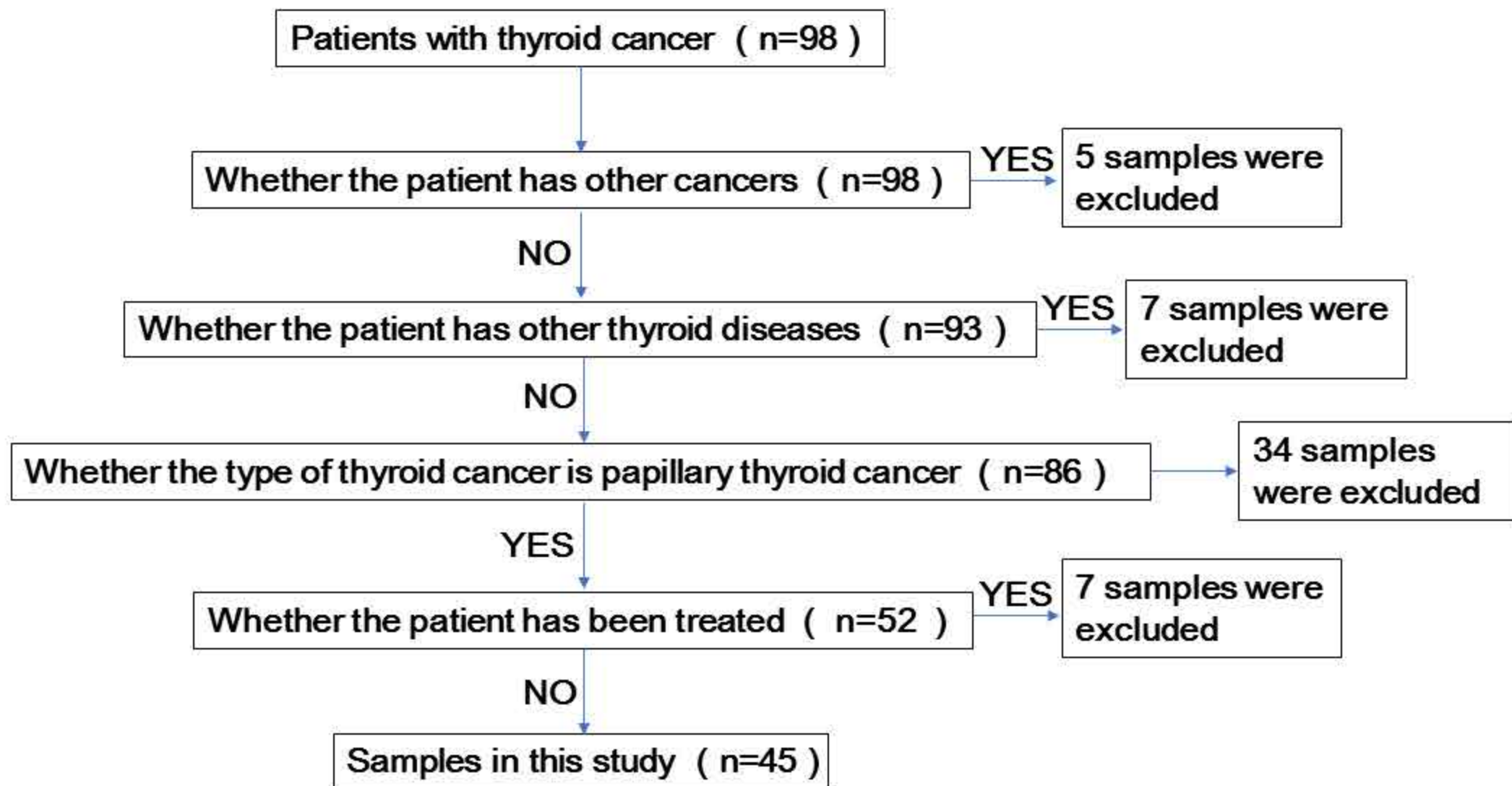

Supplement: Supplementary Materials — Figure S1: the diagram of the screening of included PTC patients. Figure S2: the selection of the potential targeted miRNAs of circLDLR. [file 3977189.f1.zip › Fig S1-revised.pdf]

## TPC-1

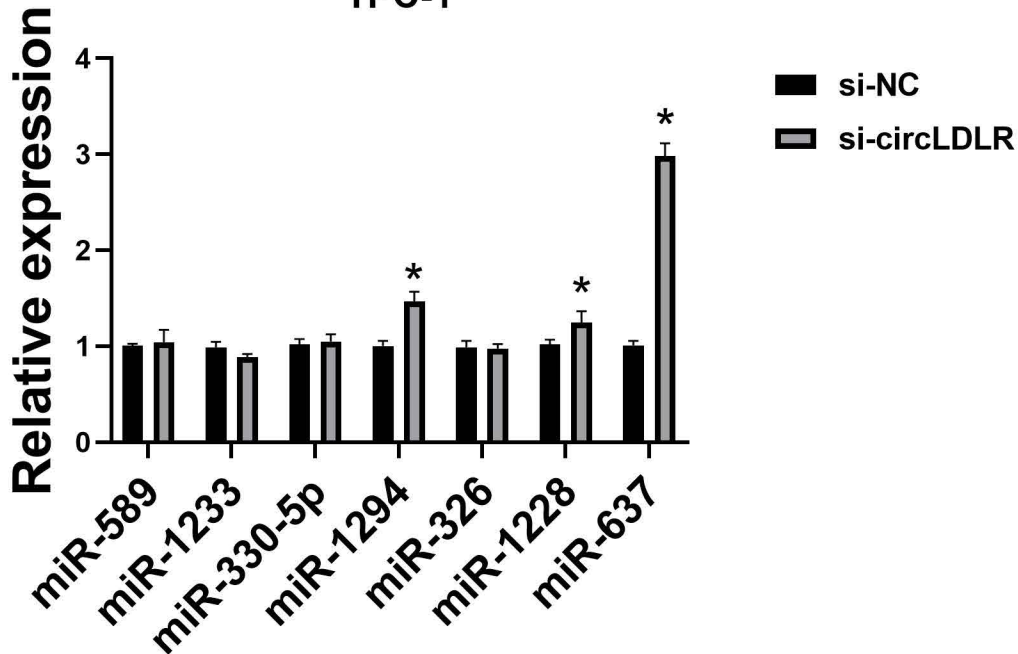

Supplement: Supplementary Materials — Figure S1: the diagram of the screening of included PTC patients. Figure S2: the selection of the potential targeted miRNAs of circLDLR. [file 3977189.f1.zip › Fig S2.pdf]
